# Neisseria meningitidis serogroup X polysaccharide as a qualified antigen for multivalent meningococcal conjugate vaccines

**DOI:** 10.3389/fimmu.2026.1943863

**Published:** 2026-10-08

**Authors:** Dan Fu, Kang Li, Xianhua Shi, Fangming Zhang, Jia Zheng, Jia Yu, Zhaoyang Xue, Shunkai Gao, Chuanzhen Lu, Ganggang Sun, Ruichuang Qian, Xinxing Long, Wensheng Xie, Lei Gao

**Affiliations:** 1 Beijing Zhifei Lvzhu Biopharmaceut Co., Ltd., Beijing, China; 2 State Key Laboratory of Drug Regulatory Science, National Health Commission (NHC) Key Laboratory of Research on Quality and Standardization of Biotech Products, National Institutes for Food and Drug Control (NIFDC), Beijing, China; 3 State Key Laboratory of Organic-Inorganic Composites, College of Life Science and Technology, Beijing University of Chemical Technology, Beijing, China

**Keywords:** MenX, MenX PS, physicochemical properties, immunogenicity, MMCVs

## Abstract

**Background:**

Meningococcal meningitis remains a severe global health threat to infants, with multivalent vaccination serving as the most effective preventive measure. In 2021, World Health Organization (WHO) released *Defeating Meningitis by 2030: A Global Roadmap*, highlighting an urgent demand for conjugate vaccines covering an expanded spectrum of meningococcal serogroups. Accordingly, the development of affordable and globally accessible diagnostic and preventive strategies, including protection against *Neisseria meningitidis* serogroup X (MenX), has become a key public health priority.

**Methods:**

A comprehensive multi-analytical strategy was implemented, incorporating whole-genome sequencing (WGS), electron microscopy, nuclear magnetic resonance (NMR), Fourier-transform infrared spectroscopy (FTIR), Congo red staining assay, size-exclusion chromatography coupled with multi-angle laser light scattering (SEC-MALLS), thermogravimetric analysis/derivative thermogravimetry (TGA/DTG), differential scanning calorimetry (DSC), immunodiffusion assays, enzyme-linked immunosorbent assay (ELISA), and serum bactericidal assay, to systematically evaluate the genetic stability of the MenX production strain, alongside the molecular structure, physicochemical properties, antigenicity, and immunogenicity of MenX polysaccharide (MenX PS).

**Results:**

Our study demonstrated the genomic stability of MenX CMCC 29040 strain and characterized the fermentation kinetics of capsular polysaccharide (CPS) throughout fermentation. Formaldehyde inactivation efficiently promoted the release of CPS from the bacteria while preserving bacterial cellular integrity. Furthermore, micro-morphological characterization for the first time directly visualized the optimal particle size range (100–200 nm) and polydispersity profile of purified MenX PS. In aqueous solution, free MenX PS adopted a compact, globular, random-coil conformation without triple-helix structure, exhibiting distinct structural differences compared with membrane anchored CPS. In addition, quantitative thermal analysis established the thermodynamic threshold of MenX PS, confirming a degradation temperature above 170 °C and superior thermal stability relative to *Neisseria meningitidis* serogroup A polysaccharide (MenA PS). Moreover, quality-qualified MenX PS exhibited specific antigenic reactivity and the MenX PS-tetanus toxoid conjugate (X-TT), prepared via reductive amination conjugation, presented robust immunogenicity and excellent batch-to-batch reproducibility. *In vivo* immunization confirmed that X-TT elicited high level MenX PS-specific IgG antibody responses and potent complement-dependent serum bactericidal activity.

**Conclusion:**

MenX PS possesses favorable physicochemical properties and specific immunogenic characteristics, supporting its eligibility as a qualified antigen component for the development of multivalent meningococcal conjugate vaccines (MMCVs).

## 1 Introduction

*Neisseria meningitidis* (*N. meningitidis*) is the causative agent of epidemic bacterial meningitis. Capsular polysaccharide (CPS) serves as a major virulence factor of pathogenic *N. meningitidis*, playing a crucial role in evading the host immune response. Among the 13 meningococcal serogroups classified according to the serological specificity of their CPS structures, six (A, B, C, W_135_, X, and Y) are associated with invasive meningococcal disease (IMD) and exhibit distinct epidemiological profiles, varying in age-group predominance and geographic distribution (1–3). Even with optimal clinical management, the case fatality rate remains high, ranging from 10% to 28%, while long-term sequelae may affect up to 36-60% of survivors (4, 5). Vaccination remains a cornerstone of public health strategy for preventing IMD. The development of meningococcal vaccines has evolved substantially from early monovalent plain polysaccharide vaccines to modern multivalent conjugate vaccines with improved immunogenicity and broader serogroup coverage (6). Currently, several licensed pentavalent conjugate vaccines targeting serogroups A, C, W_135_, and Y are commercially available, including MenQuadfi^®^ and Menactra^®^ (Sanofi) (7–9), Menveo^®^ (GSK) (10, 11), and Nimenrix^®^ (Pfizer) (12, 13). Following the initial authorization of protein-based serogroup B vaccines in the European Union (14), two pentivalent conjugate vaccines covering serogroups A, B, C, W_135_, and Y have been launched: Penbraya™ (Pfizer) (15) and Penmenvy™ (GSK) (16).

*N. meningitidis* serogroup X (MenX), first identified in the 1960s, has triggered large-scale outbreaks across the African meningitis belt, with documented epidemics in Niger, Ghana, Kenya, Burkina Faso, Togo, and Uganda (17–20). It was not until 2010 that the World Health Organization (WHO) addressed MenX in its *Weekly Epidemiological Record*, followed by its formal inclusion in the 2011 WHO position paper as a recognized cause of epidemic meningitis (21). In line with the WHO Global Roadmap *Defeating Meningitis by 2030* launched in 2021 (22), global research and development programs have increasingly prioritized multivalent conjugate vaccines incorporating serogroup X antigens. To date, the only licensed vaccine covering serogroups A, C, W_135_, Y, and X is NmCV-5^®^ from the Serum Institute of India (23, 24).

MenX PS was first isolated and purified in the 1970s (25, 26). Structural elucidation via nuclear magnetic resonance (NMR) spectroscopy subsequently confirmed that MenX PS is a homopolymer composed of (α1→4)-linked-*N*-acetyl*-D-*glucosamine-1-phosphate repeating units and lacks *O*-acetylation *(*27–29). In contrast to the sialic acid-based capsules characteristic of other pathogenic serogroups (e.g., B, C, W_135_, and Y), MenX PS consists of repeating units connected by anomeric phosphodiester bonds, exhibiting structural similarity to *N. meningitidis* serogroup A polysaccharide (MenA PS). Nevertheless, purified MenX PS demonstrates superior thermal stability relative to MenA PS (30, 31). The molecular mechanism of MenX PS biosynthesis has been elucidated. The genes responsible for polysaccharide synthesis, transport, and cell surface translocation are clustered within a single chromosomal locus designated *cps*, which is divided into six regions: A-D, D′, and E. Region A, B, and C are associated with CPS biosynthesis. Within Region A, three genes designated as *csxA*, *csxB*, and *csxC* are unique to MenX and encode enzymes essential for CPS synthesis, forming the molecular basis for PCR-based identification of serogroup X (2, 32). Genes in regions B and C are conserved across serogroups and mediate the translocation of high-molecular-weight polysaccharides to the cell surface, whereas those in Region A exhibit substantial sequence diversity (2).

In this study, quality-qualified MenX PS intended for the production of multivalent meningococcal conjugate vaccines (MMCVs) was successfully purified through commercial-scale fermentation. Whole-genome sequencing (WGS) confirmed the genetic stability of CPS biosynthesis related genes throughout fermentation, establishing a molecular foundation for accurate and efficient biosynthesis of the target polysaccharide. Capsular thickness was systematically monitored using transmission electron microscopy (TEM) throughout fermentation, providing the first direct visualization of the dynamic capsular development in MenX through the initial, logarithmic, and stationary growth phases. The currently adopted fermentation, inactivation, and centrifugation protocols effectively preserved bacterial structural integrity, whereas formaldehyde inactivation served as a critical procedure to facilitate CPS release. Comprehensive physicochemical characterization demonstrated that purified MenX PS retained canonical molecular structural features (27–29), possessed a weight-average molecular mass (Mw) approximately 10^5^ g·mol^-1^ (30, 31), adopted globular random-coil conformation without triple-helix structure in aqueous solution, and exhibited notable surface topological heterogeneity along with superior thermal stability. Serological assays verified the favorable antigenicity of MenX PS, as evidenced by specific precipitin lines generated with reference anti-MenX rabbit antiserum. Encouragingly, the covalently coupled MenX PS-tetanus toxoid conjugate (X-TT) elicited potent *in vivo* protective immune responses, establishing an immunological basis for vaccine application. Overall, these findings provide insightful evidence and technical support for the research, development, and large-scale manufacturing of MMCVs.

## 2 Materials and methods

### 2.1 Bacterial fermentation

The MenX CMCC 29040 strain was obtained from the National Institutes for Food and Drug Control (NIFDC, China). The master seed (M) was prepared by two successive subcultures of the primary seed stock, and the primary working seed (W0) was subsequently generated from the master seed via two additional subcultures. During fermentation, the bacterial strain was subjected to four serial subculture passages: primary working seed resuscitation (W1), secondary cultivation (W2), 30L-scale fermentation (W3), and 100L-scale fermentation (W4). Bacterial samples collected at the end of the 30L and 100L fermentation stages (W3 and W4) were submitted to BGI Genomics for genomic DNA extraction and whole-genome resequencing. In parallel, systematic phenotypic characterization covering cultural, morphological, biochemical, and serological properties was performed on the master seed (M), primary working seed (W0), and the final fermentation seed (100L-scale fermentation, W4). Additionally, bacterial samples were collected at five critical time points during the 100L fermentation process: initial growth phase, logarithmic growth phase, stationary phase (harvest-stage), post-formaldehyde inactivation, and inactivated bacterial pellets. All specimens were prefixed and then transferred to the Biomedical Testing Center of Tsinghua University for further processing and transmission electron microscopy (TEM) morphological analysis.

### 2.2 Genetic stability analysis of capsular polysaccharide biosynthesis genes during fermentation

Using the MenX reference genome retrieved from the NCBI database (GenBank: HF562988) (2) as a baseline, CPS biosynthesis genes located in Region A (*csxA, csxB, csxC*), Region B (*ctrE, ctrF*), and Region C (*ctrA, ctrB, ctrC, ctrD*) were accurately identified and extracted from WGS data of bacterial samples collected from distinct fermentation seed generations. Multiple sequence alignment of these target genes between the master seed (M) and serially passaged fermentation seed generations was subsequently performed using ClustalW and visualized with ESPript (33). Genomic-level characterization efficiently evaluated the genetic stability of the MenX strain across serial production subculture passages.

### 2.3 Characterization of bacterial capsular thickness

Bacterial samples collected at the five time points as described in section 2.1 were prefixed with 2.5% glutaraldehyde (Solarbio) at 4 °C for 12 hours and postfixed with 1% OsO_4_ (Sigma) at 4 °C for 1 hour. After fixation, the specimens were subjected to serial dehydration using gradient ethanol concentrations (50%, 70%, 90%, and 100% v/v), infiltrated with a 1:1 mixture of SPI-Pon™ 812 resin (SPI, USA) and acetone for 1 hour at room temperature, and embedded in pure resin for 2 hours. Resin-embedded blocks were subsequently polymerized at 60 °C for 48 hours. Ultrathin sections (50–70 nm) were prepared using a Leica Ultracut microtome, mounted onto copper grids, and stained with 2% uranyl acetate (Beijing Zhongjingkeyi Technology Co., Ltd.). Following washing and drying, stained sections were imaged using a HT-7800 TEM (Hitachi, Japan). Capsular thickness was quantified from digital micrographs with Nano Measurer 1.2 software, and all statistical analyses were completed in Origin 2025b.

### 2.4 Purification of capsular polysaccharide

MenX PS was extracted from the MenX CMCC 29040 strain according to the method described by Yang and Jennings (34). Briefly, hexadecyltrimethylammonium bromide (CTAB, Sigma) was added to the supernatant of the formaldehyde inactivated bacterial culture at a final concentration of 0.5–1.5% (w/v) to precipitate crude polysaccharides. The resulting complex polysaccharide precipitate was then dissolved in calcium chloride solution. Nucleic acid impurities were removed by ethanol precipitation at a final concentration of 25% (v/v), after which the target MenX PS was subsequently precipitated using 75–80% ethanol. Following additional phenol extraction of crude MenX PS, ultrafiltration was carried out with a 30 kDa molecular weight cut off (MWCO) membrane cassette (Millipore) to eliminate process related impurities. The retentate was precipitated again with 75–80% ethanol, washed with absolute ethanol and anhydrous acetone, and subjected to organic solvent volatilization to yield purified solid MenX PS (**Supplementary Table 1**).

### 2.5 Nuclear magnetic resonance spectroscopy analysis

The chemical structure and purity of the purified MenX PS were analyzed by ¹H, ¹³C, DEPT135, and ³¹P NMR spectroscopy. ¹H-¹H COSY (correlation spectroscopy) was employed to identify scalar couplings between adjacent protons, ¹H-¹³C HSQC (heteronuclear single-quantum coherence) spectra provided direct ¹H-¹³C correlations, and ¹H-¹³C HMBC (heteronuclear multiple-bond correlation) spectra revealed information on long-range ¹H-¹³C couplings (30, 35). Prior to measurement, purified MenX PS was lyophilized twice from D_2_O (Sigma), dissolved in 550 μL of D_2_O, and transferred to a 5 mm NMR tube. All NMR spectra were acquired on an AVANCE NEO 600 MHz spectrometer at 25 °C. Chemical shifts were referenced to previously published ¹H and ¹³C NMR datasets of MenX PS to confirm structural integrity (29). The *O*-acetylation levels were also assessed, given its critical impact on the immunogenicity of meningococcal polysaccharides-based vaccines.

### 2.6 Fourier-transform infrared spectroscopy analysis

A 5 mg portion of purified MenX PS was mixed with potassium bromide (KBr) and pressed into a 1 mm-thick pellet. An FTIR spectrum was recorded on a Nicolet 8700 spectrophotometer (Thermo Fisher Scientific, USA) in the wavenumber range from 4000 to 500 cm^-^¹, with a resolution of 2 cm^-^¹ over 40 accumulated scans.

### 2.7 Congo red staining assay

Congo red staining assay was carried out to evaluate the chain conformation of MenX PS in aqueous solution. A bathochromic red shift of the maximum absorption wavelength (λ_max_) is a characteristic signature of polysaccharide triple-helix conformation (36, 37). Briefly, equal-volume aliquots (1 mL each) of purified MenX PS solution (1 mg·mL^-1^) and Congo red (Solarbio) stock solution (80 μM) were mixed homogeneously by vortexing. Subsequently, NaOH solution (1 M) was added to the mixture to generate a series of final NaOH concentration gradients ranging from 0 to 0.5 M. Visible light absorption spectra were recorded over a range of 400–600 nm at room temperature using a spectrometer, with the Congo red blank solution serving as the negative control.

### 2.8 Scanning electron microscopy analysis

A 5 mg portion of purified MenX PS was affixed to a sample stub with conductive carbon tape and allowed to air-dry completely. Images were acquired using a JSM-7800F thermal field-emission SEM (JEOL, Japan) at an accelerating voltage of 10.0 kV under magnifications of 22,000× and 75,000×.

### 2.9 Transmission electron microscopy analysis

A 5 μL aliquot of purified MenX PS was loaded onto a freshly glow-discharged carbon-coated grid (300 mesh, Beijing Zhongjingkeyi Technology Co., Ltd.). After incubating for 1 minute, excess liquid was blotted away, and the grid was stained with 2% (w/v) uranyl acetate solution for 40 seconds. Excess staining solution was aspirated off, and the grids were dried at room temperature. Images were acquired using a HT 770 TEM (Hitachi, Japan) operating at 100 kV, and equipped with a 4 K × 4 K Ultrascan CCD camera, at magnifications of 20,000×, 40,000×, and 60,000×.

### 2.10 Polysaccharide molecular mass determination

The absolute molecular mass of the purified MenX PS was determined by size-exclusion chromatography (SEC) coupled with refractive index (RI) in line with multi-angle laser light scattering (MALLS) detectors, as previously described (38). Analyses were conducted at 25 °C using a Wyatt WTC-030S5 column (Wyatt Technology). Elution profiles were simultaneously monitored using an Optilab rEX refractive index detector (Wyatt Technology) for concentration determination and a DAWN II MALLS detector (Wyatt Technology) for light scattering signals measurement. Triplicate injections were performed, and molecular mass parameters were calculated based on averaged analytical data. The specific refractive index increment (dn/dc) was fixed at 0.15 mL·g^-1^. The weight-average molar mass (Mw) and polydispersity index (PDI, Mw/Mn) were calculated according to the Zimm fitting formalism.

### 2.11 Thermogravimetric analysis/derivative thermogravimetry analysis

Thermal analysis of MenX PS and MenA PS were performed on a simultaneous thermal analyzer (TGA/DSC3+, METTLER) under a continuous nitrogen atmosphere. Approximately 5 mg of each sample was loaded into an alumina crucible and sealed, while an empty crucible served as the blank reference. Samples were heated from room temperature to 600 °C at a constant heating rate of 3 °C·min^-^¹.

### 2.12 Differential scanning calorimetry analysis

The DSC curve of MenX PS was obtained using DSC1 (Mettler-Toledo, Switzerland) calibrated with an indium standard (156.6 ± 0.3 °C). Approximately 5 mg of purified MenX PS was precisely weighed into an aluminum pan, hermetically sealed, and heated from -50 to 300 °C at a constant heating rate of 2 °C·min^-^¹. The entire test process was maintained under a dry nitrogen atmosphere with a continuous flow rate of 50 mL·min^-1^.

### 2.13 Antigenicity assay

Double immunodiffusion assay (39): A 1% (w/v) agarose solution was prepared in pre-heated sterile saline (0.85% NaCl) and poured onto flat glass microscope slides after cooling to form the gel matrix. Wells with a diameter of 3 mm were punched in the gel matrix with a center-to-periphery spacing of 3–5 mm. The central well was loaded with 10 μL of MenX PS antigen solution, while the peripheral wells were individually loaded with 10 μL of standard rabbit antisera (NIBSC) specific to meningococcal serogroups A, C, W_135_, Y, and X, respectively. Sterile saline served as the negative control. Subsequently, the agar plates were then placed in a humid chamber and incubated at 37 °C for 16 hours, and specific immunodiffusion was visualized after staining.

Single radial immunodiffusion assay: A 1% (w/v) agarose saline solution was cooled to approximately 56 °C and mixed with a predetermined volume of specific anti-MenX rabbit antiserum. The mixture was immediately poured onto glass microscope slides and left to solidify at room temperature. Triplicate 3 mm-diameter wells were punched in the solidified gel, and each well was loaded with 10 μL of MenX PS or X-TT conjugate aqueous solution. The agar plates were then incubated in a humid chamber at 37 °C for 16 hours. After staining, the diameters of specific immunodiffusion precipitin rings were measured for the semi-quantitative assessment of antigenic potency.

### 2.14 Glycoconjugate preparation

Purified MenX PS was dissolved in ultrapure water. The polysaccharide was activated by adding 1-cyano-4-dimethylaminopyridinium tetrafluoroborate (CDAP, Sigma) solution at a 1:1 mass ratio, and the reaction pH was strictly maintained at 10.0–11.0 for 30 minutes. Subsequently, an equal-mass solution of adipic dihydrazide (ADH, Sigma) was supplemented, and the mixture was incubated at pH 8.0–9.0 for 30–60 minutes to synthesize MenX-PS-ADH derivatives. The derivatized solution was ultrafiltered to remove small-molecule impurities, including residual CDAP and ADH. Purified tetanus toxoid (TT) was added at an approximate 1:1 polysaccharide to protein mass ratio. Conjugation was performed in the presence of 1-ethyl-3-(3-dimethylaminopropyl) carbodiimide (EDAC, Sigma) at pH 5.0–6.0 for 6 hours, and the reaction was terminated by adjusting the pH to approximately 7.0. After incubation, the reaction system was ultrafiltered using a 100 kDa MWCO membrane cassette (Millipore) to eliminate residual EDAC. The final retentate was collected to obtain three batches of X-TT glycoconjugates (**Supplementary Table 2**).

### 2.15 Immunization

Animal experiments were conducted in strict accordance with the guidelines of the Institutional Animal Care and Use Committee (IACUC). Six-week-old specific-pathogen-free (SPF) female ICR mice were purchased from SPF (Beijing) Biotechnology Co., Ltd. and housed in ventilated cages under environmentally controlled conditions at Beijing Zhifei Lvzhu Biopharmaceutical Co., Ltd. (12 hours light/dark cycle, 21 °C, 50% humidity). For immunogenicity study, mice were randomly divided into four groups (Control, X-TT batch1, X-TT batch2, and X-TT batch3), each comprising ten mice. The immunization regimen was designed as a primary immunization followed by a booster injection with a two-week interval. For each dose, 2.5 μg of X-TT conjugate dissolved in 500 μL sterile saline was subcutaneously administered to each mouse, while the control group received an equal volume of sterile saline via subcutaneous injection. At week 2 following booster immunization, mice were euthanized via intraperitoneal injection of an overdose of pentobarbital sodium (170 mg/kg). Once animals attained deep anesthesia accompanied by complete loss of the righting, corneal, and nociceptive reflexes, orbital sinus blood collection was performed immediately. After blood sampling, cervical dislocation was carried out to confirm irreversible death and prevent potential recovery. All procedures were performed under deep anesthesia to minimize animal distress. After standing at room temperature for 30 minutes, blood samples were centrifuged at 4 °C and 500× g for 10 minutes to separate serum from blood cells. Harvested serum samples were heat-inactivated at 56 °C for 30 minutes and utilized to determine the titers of MenX PS-specific IgG-binding antibody and serum bactericidal activity against MenX CMCC 29040 strain.

### 2.16 Enzyme-linked immunosorbent assay (ELISA)

ELISA was performed to determine serum MenX PS-specific binding antibody titers. Briefly, each well of a 96-well assay plate (Corning) was pre-coated with 100 μL of PBS solution containing MenX-BSA conjugate (covalent conjugate of MenX PS and bovine serum albumin, BSA, Sigma) at a concentration of 2 μg·ml^-1^. Plates were incubated overnight at 4 °C and then washed five times with washing buffer composed of PBS and 0.05% (v/v) Tween-20 (Solarbio). Subsequently, each well was blocked with 250 μL of blocking buffer containing PBS and 5% milk, followed by 2 hours incubation at 37 °C. After thorough washing for five cycles, serially diluted mouse serum samples were added at 100 μL per well. Serum samples were initially diluted 1:100 in blocking buffer and then subjected to two-fold serial dilutions. Then, Plates were incubated at 37 °C for 1 hour and washed five times. Next, HRP-conjugated goat anti-mouse IgG secondary antibody (Abcam) was diluted to a ratio of 1:10,000 with blocking buffer, and 100 μL of the diluted antibody solution was added to each well. After 30 minutes incubation at 37 °C, plates were washed seven times. Finally, 100 μL of TMB was added to each well, and after 10–15 minutes, the color development reaction was stopped with 50 μL of 2 N sulfuric acid. The resulting plate readouts were measured at a wavelength of 450 nm to calculate the corresponding endpoint binding antibody titers.

### 2.17 Serum bactericidal assay

Given that the primary clearance mechanism of *Neisseria meningitidis* in humans relies on specific antibody-dependent and complement-mediated bactericidal activity (40), serum bactericidal assay was employed to evaluate the functional immunogenic potency of the X-TT conjugates. The MenX CMCC 29040 strain was streaked for isolated colonies and cultured overnight at 37 °C with 5% CO_2_ on agar plates. Colonies were subcultured in shake flasks and incubated at 37 °C with 5% CO_2_ for 3–4 hours until logarithmic growth phase. Subsequently, the bacteria were resuspended in modified D-PBS (Dulbecco’s PBS containing 0.5 mM MgCl_2_, 0.9 mM CaCl_2_, and 0.1% glucose, pH 7.4) to achieve an absorbance of 0.35 at 660 nm with a light path of 16 mm. The cell suspension was further diluted with D-PBS to a final concentration of 50–60 CFU·12.5 μL^-1^. Heat-inactivated mouse serum samples were serially two-fold diluted (starting dilution at 1:8) in flat-bottom 96-well tissue culture plates (Costar, Inc., Cambridge, MA, USA) using D-PBS. An equal volume of bacterial suspension was gently mixed with complement serum derived from 3–4 weeks-old juvenile rabbits (Pel Freeze, Inc., Brown Deer, WI, USA), and 25 μL of the bacteria-complement mixture was added to the serially diluted serum, yielding a final reaction volume of 50 μL in each well. Plates were then incubated at 37 °C with 5% CO_2_ for 60 minutes. Subsequently, 100 μL molten tryptic soy broth (TSB, Sigma) containing 0.9% agar and 1% tetrazolium red (TTC, Sigma) was added to each well, followed by overnight incubation at 37 °C under 5% CO_2_. The bactericidal titer for each serum sample was expressed as the reciprocal of the highest serum dilution that yielding ≥50% killing compared to the number of target cells present before incubation with serum and complement (40).

### 2.18 Statistical analysis

All treatments and assays were performed in triplicates and the data were represented by their mean ± standard deviation (SD). Statistical significance of differences between groups was analyzed by a one-way analysis of variance (VNOVA). Data were analyzed using Origin 2025b and Prism 10.

## 3 Results

### 3.1 Genetic stability of the bacterial strain during fermentation

Phenotypic characterization was performed on the master seed (M), primary working seed (W0), and the final fermentation seed (W4) of the MenX strain. No bacterial growth was observed at 25 °C for all three seed generations. Under cultivation conditions of 35–37 °C and 5–10% CO_2_, all strains yielded typical meningococcal colonies with consistent morphological features: colorless, circular, raised, smooth, and either translucent or semi-transparent with a dew-drop appearance. Gram staining confirmed that the strains from all seed generations were Gram-negative cocci, predominantly occurring as diplococci or individual cocci, with no extraneous microbial contamination detected under microscopic observation. Biochemical assays verified consistent physiological characteristics, including acid production from glucose and maltose without gas formation. Serological agglutination tests demonstrated that all three seed generations exhibited robust specific reactivity with specific anti-MenX rabbit antiserum, forming obvious granular agglutinates with a titer of 1:160, which corresponded to half the potency of the original standard antiserum (1:320), thereby confirming stable CPS biosynthesis and precise serogroup identity (**Supplementary Figure 1**). Overall phenotypic evidence indicated that the MenX maintained intact and stable strain-specific biological properties throughout the entire seed amplification and industrial fermentation from master seed to final working seed.

To further validate the genetic consistency of strains during fermentation, WGS was performed on bacterial isolates from the W3 and W4 fermentation stages. Consistent with previous reports, all key genes responsible for CPS biosynthesis within the MenX *cps* locus, including *csxA*, *csxB*, *csxC*, *ctrE*, *ctrF*, *ctrA*, *ctrB*, *ctrC*, and *ctrD*, were accurately identified and retrieved from the WGS datasets of both fermentation stages (**Supplementary Table 3**). Genetic composition, gene copy number, and organizational arrangement in Region A further confirmed that the tested strains belonged to the MenX genotype (2) (**Figure 1A**). Multiple sequence alignment of CPS biosynthesis-related genes across sequential seed and fermentation stages, including the master seed (M), primary working seed (W0), and industrial fermentation seeds (W3 and W4), exhibited 100% nucleotide sequence identity (**Figure 1B**; **Supplementary Figures 2**, **3**). Collectively, these molecular genetic results confirmed that the established fermentation manufacturing process reliably maintains high genetic stability of the MenX strain throughout production scale-up.

**Figure 1:**
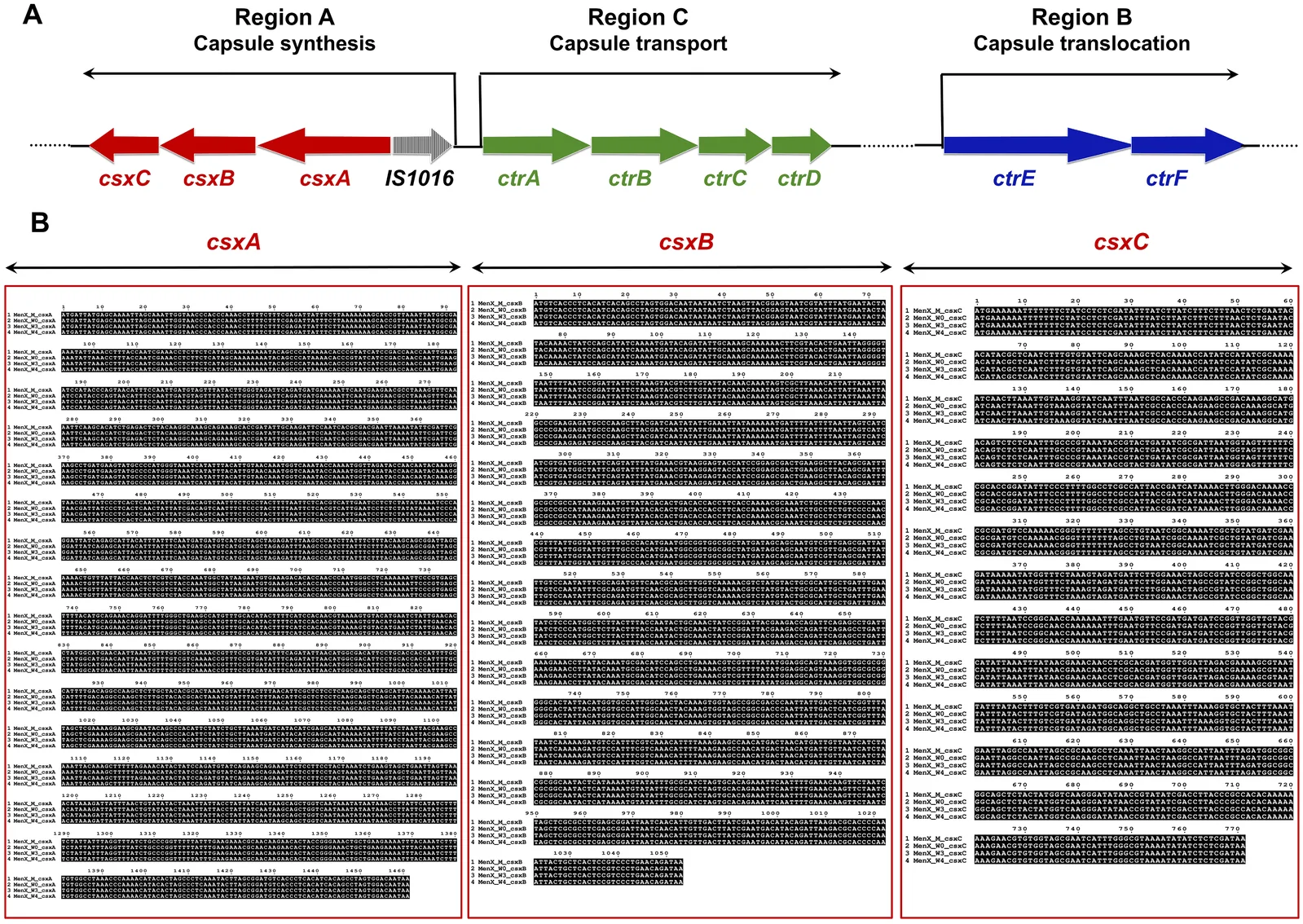
Genetic stability analysis of the capsule synthesis genes during fermentation. **(A)** Schematic of the gene cassette within the *cps* locus of *Neisseria meningitidis* serogroup X (MenX) CMCC 29040 strain, depicting genes involved in capsule synthesis, translocation, and transport. Arrows indicate individual genes, with lengths proportional to gene size and orientations reflecting transcriptional direction. **(B)** Multiple sequence alignment of the capsule synthesis genes (*csxA*, *csxB*, *csxC*) in Region A across the fermentation process. MenX_M, master seed; MenX_W0, primary working seed; MenX_W3, 30 L-scale fermentation; MenX_W4, 100 L-scale fermentation.

### 3.2 Fermentation process monitoring

TEM was performed to observe bacterial ultrastructures across key fermentation and post-treatment stages, including the initial, logarithmic, and stationary growth phases, as well as post-formaldehyde inactivation and inactivated pellets. TEM observations demonstrated that bacterial cells retained intact morphological structures throughout the entire manufacturing process, with no significant cell rupture or autolysis detected at all fermentation stages. These findings indicated that the MenX CMCC 29040 strain possesses robust structural stability under established process parameters. Such stable cellular morphology effectively prevents the leakage of intracellular contaminants, including nucleic acids, proteins, and endotoxins, thereby minimizing potential interference with downstream polysaccharide purification (**Figure 2A**).

**Figure 2:**
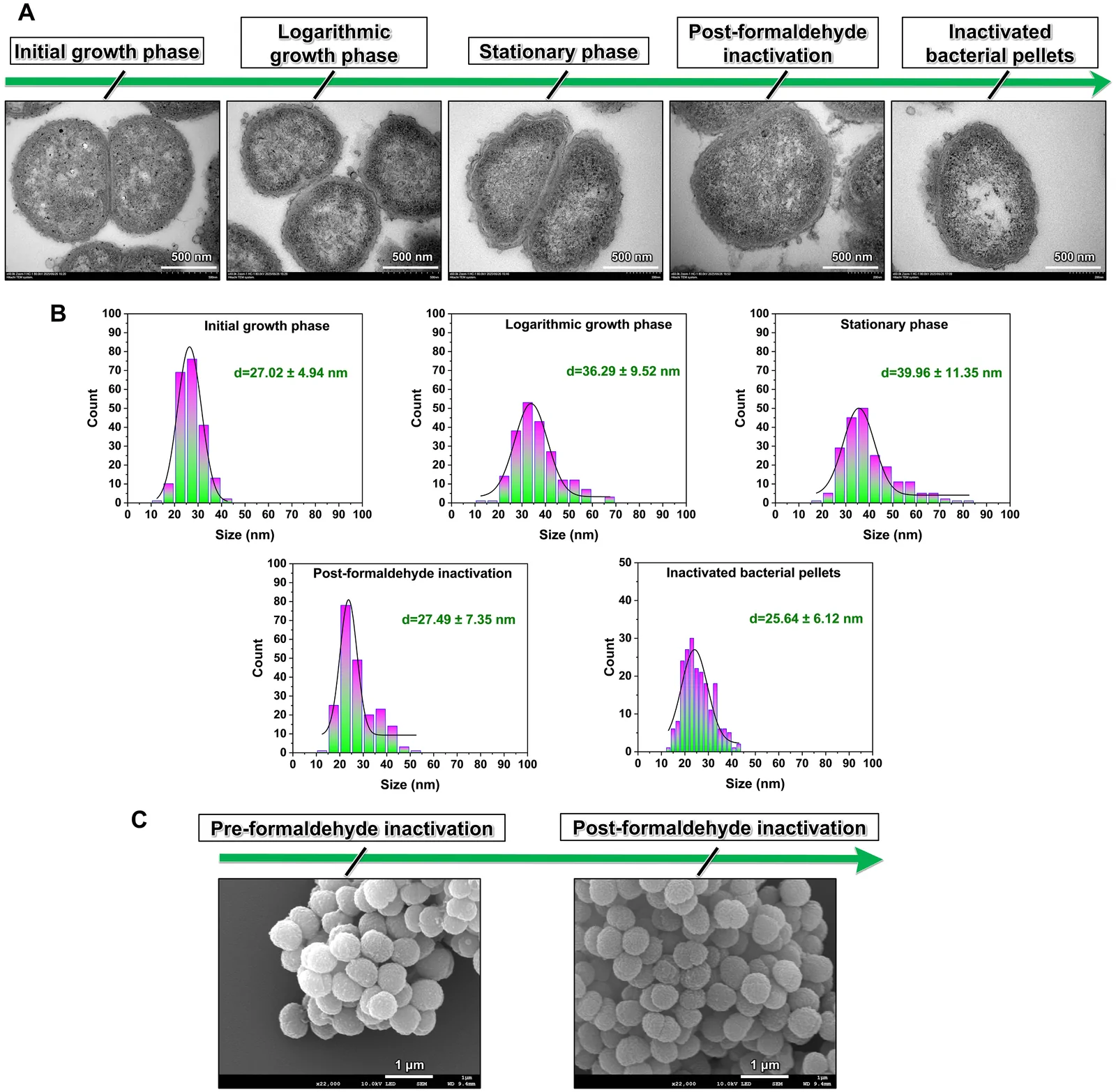
Effects of fermentation and inactivation on cellular integrity and capsular thickness of MenX. **(A)** Transmission electron microscopy (TEM) images of bacterial sections at different fermentation stages, post-formaldehyde inactivation, and inactivated bacterial pellets. **(B)** Histogram illustrating the distribution of capsular thickness. Statistical quantification of capsular thickness corresponding to the groups shown in **(A)**. Bacterial capsules were relatively thin at the early fermentation stage, gradually thickened during cultivation, and remained stable at later fermentation phases. No obvious structural deformation was observed following formaldehyde inactivation and centrifugation. **(C)** Scanning electron microscopy (SEM) images of overall bacterial morphology pre- and post-formaldehyde inactivation. No bacterial lysis or membrane rupture was detected under either condition.

Dynamic quantification of capsular thickness revealed a progressive increase during bacterial cultivation, consistent with active CPS biosynthesis during cell growth. The average capsular thickness was 27.02 ± 4.94 nm at the initial growth phase, increased to 36.29 ± 9.52 nm during the logarithmic growth phase, and further elevated 39.96 ± 11.35 nm at the stationary phase (pre-formaldehyde inactivation). Following formaldehyde inactivation treatment, the average capsular thickness decreased significantly to 27.49 ± 7.35 nm, corresponding to an approximate reduction of 13 nm. Meanwhile, an increased number of non-bacterial globular particles was observed in TEM fields of view. Combined with the previously identified random-coil conformation of MenX PS in aqueous solution, these results suggested that formaldehyde inactivation induced partial capsule dissociation and released free CPS fragments into the culture medium. Subsequent centrifugation of inactivated bacterial pellets caused a slight further reduction in average capsular thickness to 25.64 ± 6.12 nm. TEM imaging confirmed that centrifugation did not damage bacterial cellular integrity and only marginally contributed to polysaccharide release (**Figure 2B**).

To comprehensively assess the impact of formaldehyde inactivation on bacterial structural integrity, SEM was applied to supplement conventional TEM ultrastructural observations. SEM morphological analysis showed no detectable cell wall rupture, perforation, or cytoplasmic leakage in bacteria pre- and post-formaldehyde inactivation. These findings indicated that the current inactivation parameters (formaldehyde concentration and treatment duration) are sufficiently mild to effectively inactivate pathogens while preserving intact cellular integrity (**Figure 2C**). Collectively, microscopic evidence demonstrated that the optimized inactivation process minimizes the release of intracellular impurities, ensuring stable manufacturing performance and consistent quality of the final MenX PS.

### 3.3 Structural characterization and identification of Neisseria meningitidis serogroup X polysaccharide

The chemical structure of the MenX PS was elucidated via NMR spectroscopy. The ¹H NMR spectrum exhibited a characteristic anomeric proton signal of glucose at δ 5.58 ppm. Combined with homonuclear and heteronuclear coupling constants, this signal confirmed the α-configuration of the glycosidic bond (41). Within the pyranose ring proton region (δ 4.10–3.86 ppm), six sugar ring protons (H2-H6) were unambiguously assigned based on two-dimensional NMR datasets. The ¹³C NMR spectrum displayed distinct signal splitting at C1, C2, C4, and C5 positions, which originated from long-range coupling with ³¹P nuclei, indicating these carbon atoms in close proximity to the phosphodiester group. Through-bond connectivity from C1 to C6 was established via ¹H-¹H COSY correlations (H1-H2, H2-H3, H3-H4, H4-H5, H5-H6) and confirmed by long-range heteronuclear correlations in the ¹H-¹³C HMBC spectrum. A key ^1^H-^13^C HMBC correlation between H5 and C1, together with a negative C6 signal and the absence of a C7 signal in the DEPT135 spectrum, verified the formation of a sugar ring from C1 to C5. A characteristic acetyl proton (H8) was detected at δ 2.09 ppm. Long-range correlations between C7 and both H8 and H2 indicated covalent attachment of an N-acetyl group at the C2 position of each glycosyl repeating unit. Collectively, multi-dimensional NMR data, including edited ¹H-¹H COSY ^1^H-^13^C HSQC, and ^1^H-^13^C HMBC spectra, demonstrated that MenX PS is a linear, unbranched polysaccharide with a repeating unit defined as (α1→4)-linked-*N*-acetyl-*D*-glucosamine-1-phosphate (28, 29). Furthermore, no diagnostic *O*-acetylation peak was detected in the ^1^H-^13^C HSQC spectrum, excluding the existence of *O*-acetylation modification (**Figure 3A**, **Supplementary Figure 4**; **Supplementary Table 4**).

**Figure 3:**
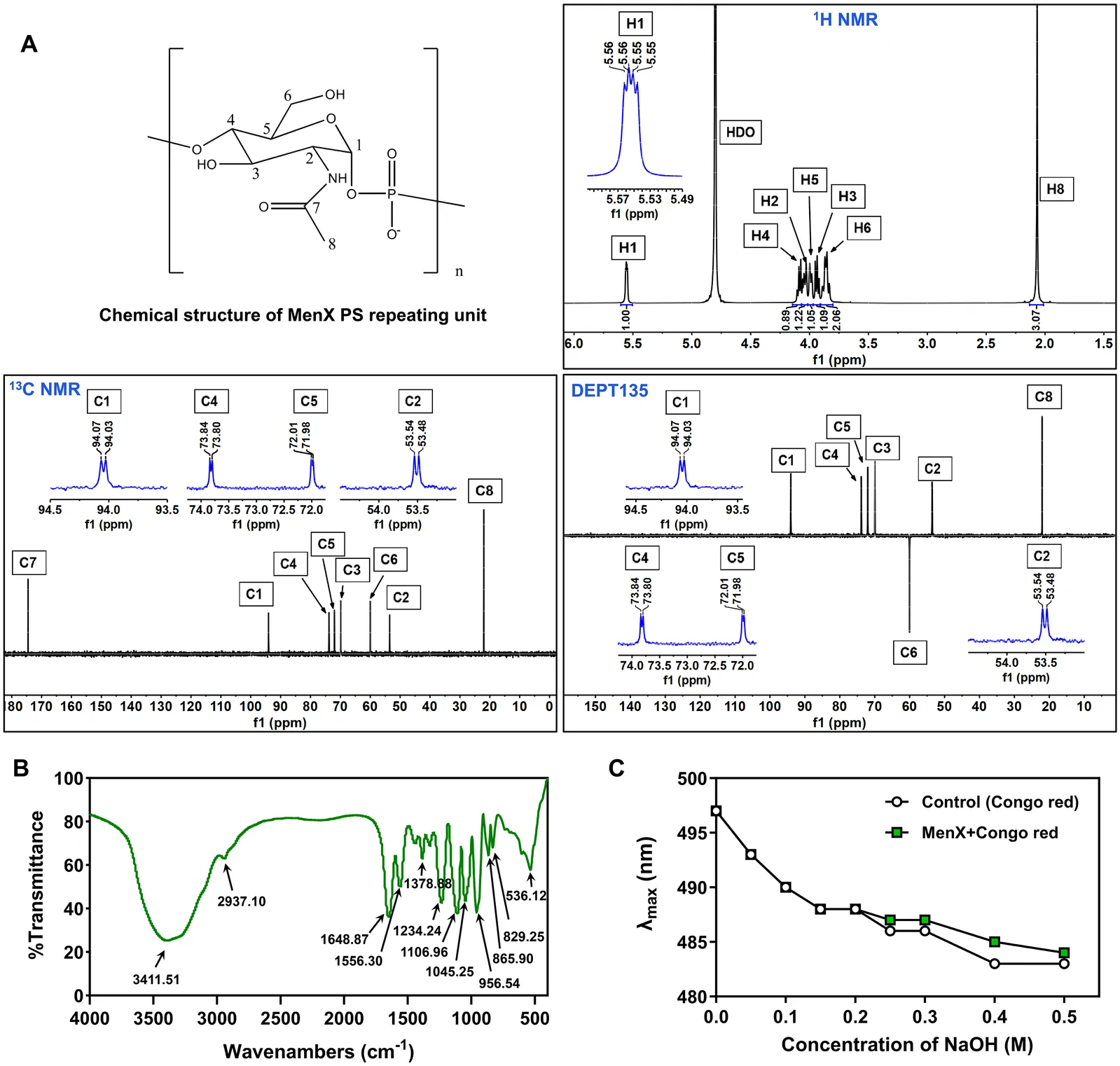
Structural characterization of *Neisseria meningitidis* serogroup X polysaccharide (MenX PS). **(A)** Nuclear Magnetic Resonance (NMR) spectroscopic identification. Depicted are the chemical structure of MenX PS repeating unit and the corresponding ¹H NMR, ¹³C NMR, and DEPT135 NMR spectra. **(B)** Fourier-transform infrared (FTIR) spectroscopy analysis, showing characteristic absorption bands for MenX PS. **(C)** Higher-order structure analysis in aqueous solution. The maximum absorption wavelength (λ_max_) of Congo red and Congo red-MenX PS mixed solutions was determined across a gradient of NaOH concentrations.

FTIR was performed to verify the functional group composition of purified MenX PS. Typical characteristic absorption bands were identified in FTIR spectrum, including a broad O-H stretching vibration at 3411.51 cm^-1^, asymmetric aliphatic C-H stretching around 2937.10 cm^-1^, and representative amide group signatures comprising a C=O stretching at 1648.87 cm^-1^ and an N-H bending at 1556.30 cm^-1^. An intense band at 1234.24 cm^-1^ was assigned to the asymmetric O=P stretching of a phosphodiester linkage. Key structural features of the glycosidic configuration and pyranose ring were also identified: absorption bands near 850 cm^-1^ supported the α-glycosidic configuration, while continuous absorption signals ranging from 950 to 1050 cm^-1^ corresponded to the typical vibration of D-pyranose rings (37, 42). The absence of a characteristic absorption band at 1370 cm^-1^ ruled out the presence of uronic acid residues. Additional minor absorption bands were assigned to C-H/O-H bending at 1378.88 cm^-1^ and pyranose ring deformation vibration at 536.12 cm^-1^. Consistent with NMR results, FTIR spectroscopic analysis confirmed that the repeating unit of MenX PS is (α1→4)-linked-*N*-acetyl-*D*-glucosamine-1-phosphate (**Figure 3B**).

The higher-order structure of MenX PS in aqueous solution was further characterized to clarify its structural state (ordered helix vs. random-coil), providing fundamental guidance for the optimization of downstream conjugation procedures. Based on the Congo red staining assay, no significant red shift of the λ_max_ was observed for the Congo red-MenX PS mixture compared relative to pure Congo red solution across a NaOH concentration range of 0–0.5 M. The absence of a red bathochromic shift demonstrated that MenX PS cannot form ordered triple-helix conformation, and polysaccharide chains exclusively adopted random-coil in aqueous solution (**Figure 3C**).

### 3.4 Microstructural characterization of Neisseria meningitidis serogroup X polysaccharide

Following structural verification of the purified MenX PS, we analyzed its physical morphology in solid state using SEM. SEM images showed that MenX PS presents irregular globular particles with a polydisperse size distribution (**Figure 4A**, left panel). Higher-magnification views further revealed distinct topographical heterogeneity particle surfaces, including irregular depressions and protrusions, along with visible intermolecular cross-linking among polysaccharide particles (**Figure 4A**, right panel). Particle size distribution was statistically quantified based on over 200 randomly selected particles using Nano Measurer 1.2 software, yielding an average particle diameter of approximately 160 nm (**Figure 4B**). These morphological and dimensional profiles provided fundamental physical references for elucidating the formulation behavior and immune-interactive properties of MenX PS.

**Figure 4:**
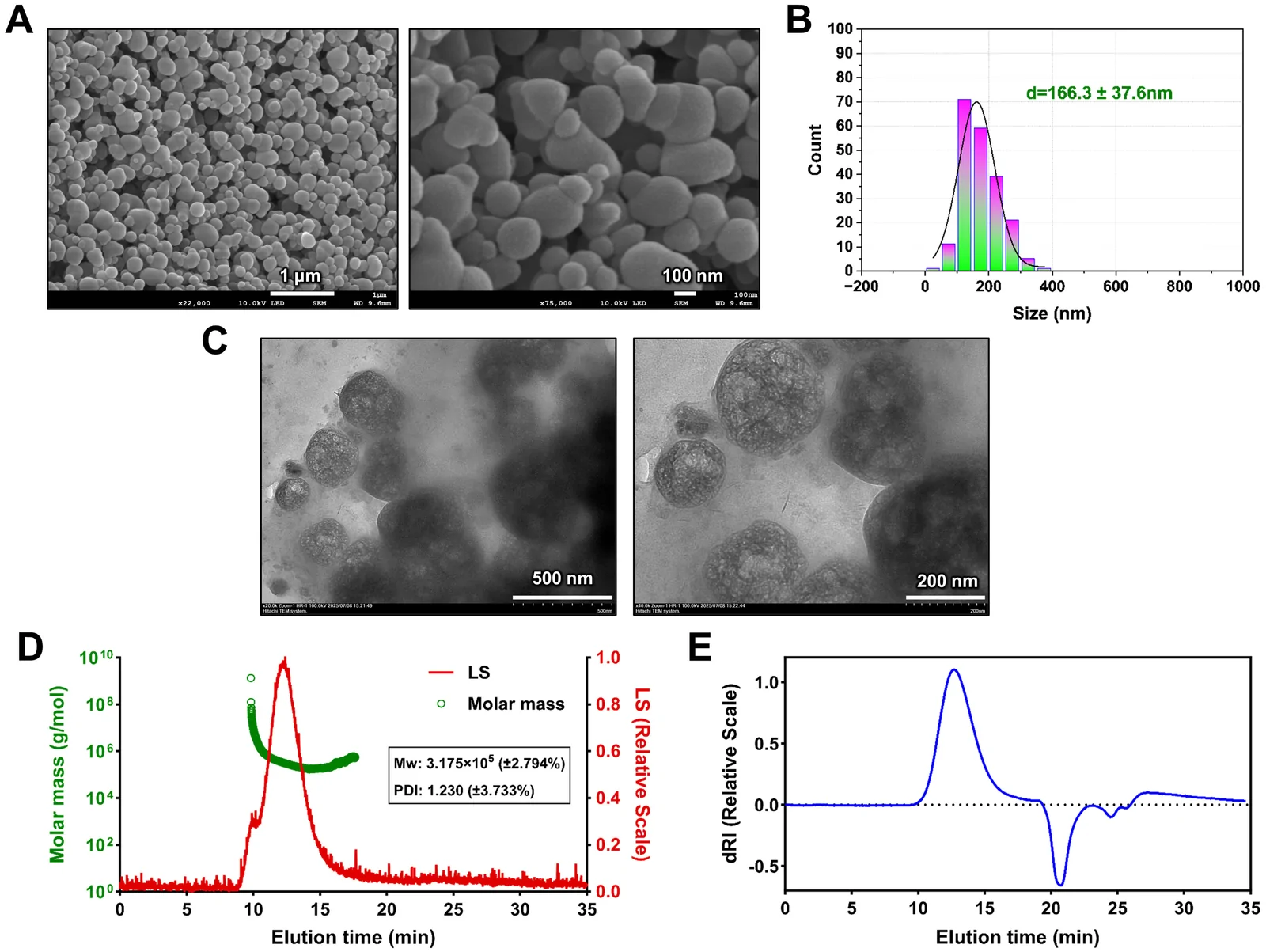
Microstructure and molecular mass analysis of MenX PS. **(A)** Solid state morphology and particle size distribution. Left panel: representative SEM image (22,000×), right panel: representative high-magnification SEM image (75,000×) showing surface topography. **(B)** Statistical histogram of particle size distribution in the solid state. **(C)** Solution state morphology analyzed by TEM. Left panel: representative TEM image (20,000×), right panel: representative high-magnification TEM image (40,000×). **(D)** Molecular mass analysis by size-exclusion chromatography with multi-angle laser light scattering (SEC-MALLS). The chromatogram shows the light scattering (LS) signal (red curve) and the corresponding molecular mass distribution at each elution volume (green dots). Mw denotes the weight-average molecular mass, and polydispersity index (PDI) represents the dispersity. **(E)** Differential refractive index (dRI) chromatographic signal.

To resolve the nanoscale structural features beyond SEM resolution limits, TEM was performed to characterize the solution conformation of MenX PS. TEM analysis revealed that MenX PS exists as densely packed globular random-coil structure with considerable polydispersity in aqueous solution, with hydrodynamic diameters ranging from 50 to 400 nm. Particles with diameters of 100–200 nm constituted the dominant population, suggesting that this size range corresponded to the thermodynamically favorable conformation. Weak non-covalent interactions among polysaccharide chains were also observed, which assembled into loose fibrous networks stabilized by hydrogen bonds and van der Waals forces. Such network structures exhibited low structural stability and readily disassembled under sonication, mechanical agitation, or dilution, ultimately dissociating into uniformly dispersed monodisperse globular particles (**Figure 4C**, left panel). High-magnification TEM imaging further revealed an irregular, rough, and porous surface microstructure (**Figure 4C**, right panel). These unique morphological traits substantially affect the solution behavior and functional performance of MenX PS. In summary, high-resolution TEM observations directly visualized the chain conformation, self-assembly pattern and surface ultrastructure, corroborated the SEM findings and provided critical ultrastructural evidence supporting the structure-function relationship of MenX PS.

SEC-MALLS analysis indicated that MenX PS eluted as a single broad chromatographic peak, with a Mw of 3.175×10^5^ g·mol^-1^ (± 2.794%) and a PDI (Mw/Mn) of 1.230 (± 3.733%). The obtained Mw was consistent with the previously reported molecular mass range of 152,000 to 512,000 g·mol^-1^ for MenX PS (30) (**Figure 4D**). The differential refractive index (dRI) chromatogram, responsive to all dissolved solutes, displayed two predominant peaks accompanied by several minor peaks. The broader primary dRI peak co-eluted synchronously with the light scattering (LS) signal, confirming this fraction as the target MenX PS. In contrast, extra low-molecular-weight dRI peaks lacked LS responses, indicating the presence of trace residual impurities, such as fragmented genomic DNA or protein debris (43) (**Figures 4D, E**).

### 3.5 Thermal stability analysis of Neisseria meningitidis serogroup X polysaccharide

Thermal stability of polysaccharide antigens is a crucial indicator for the formulation development and industrial production of conjugate vaccines. TGA/DTG was performed to evaluate the thermal decomposition behavior of MenX PS powder under programmed heating conditions. MenX PS exhibited three distinct weight loss stages during thermal treatment. The initial stage (30–170 °C, ∆m = 17.54%) was attributed to the evaporation of bound water and residual solvents (44). The second stage (171–260 °C, ∆m = 19.68%) displayed a prominent DTG peak temperature (T_peak_) at 228.45 °C, representing the primary degradation onset of polysaccharide backbones. This stage involved extensive depolymerization triggered by glycosidic bond cleavage, the loss of crystalline water, and the release of low-molecular-weight volatile byproducts generated from the breakage of C-C and C-O bonds (45). The third stage (261–600 °C, ∆m = 19.83%) corresponded to deep carbonization and pyrolysis of preliminary degradation fragments. During this process, the carbon backbone underwent structural disordering, while functional groups including carbonyl and carboxyl groups decomposed and released CO and CO_2_. Ultimately, the residual polysaccharide components were converted into stable carbonaceous residues after complete pyrolysis (**Figure 5A**).

**Figure 5:**
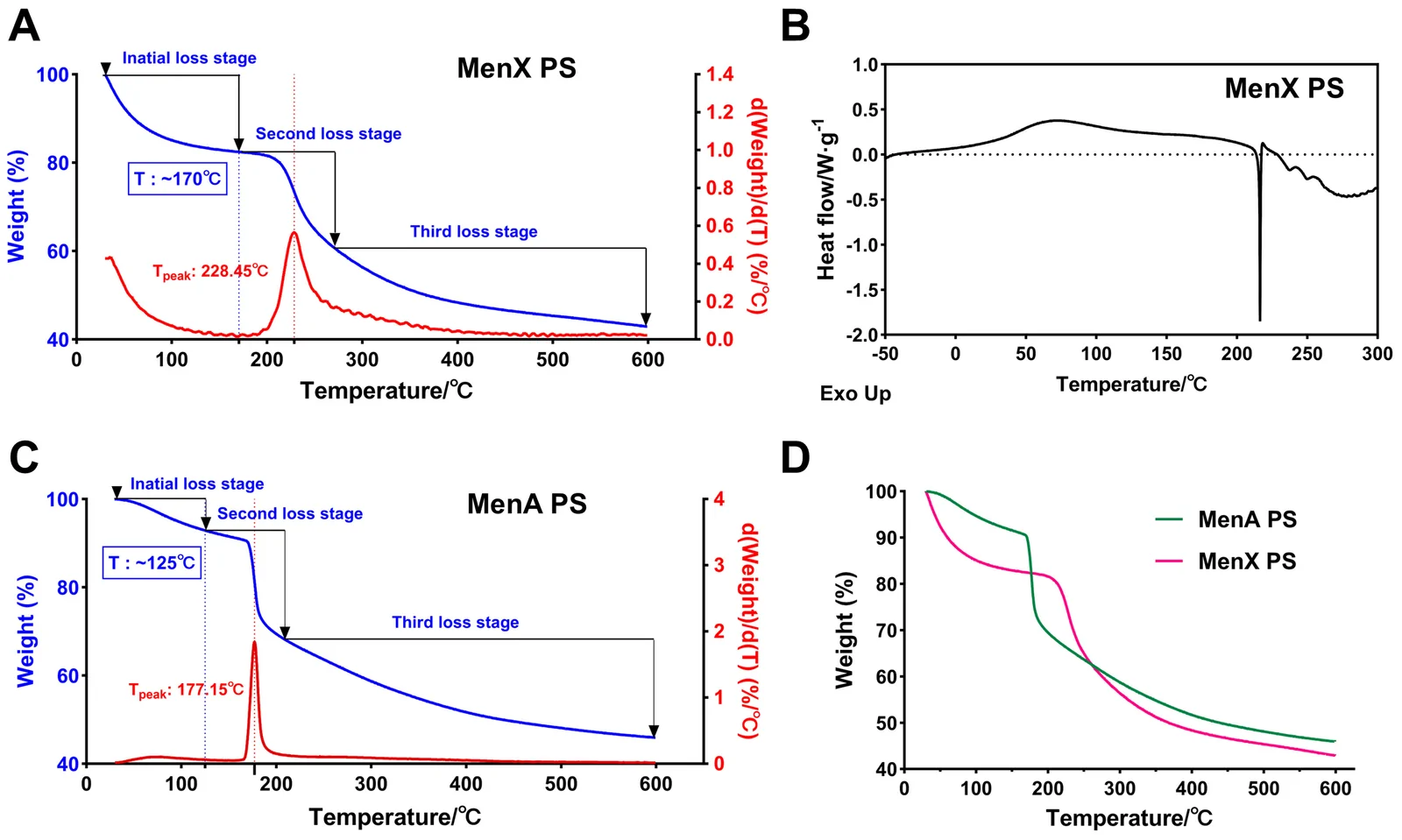
Thermal stability analysis. **(A)** Thermogravimetric/derivative thermogravimetric (TGA/DTG) curves of MenX PS obtained under nitrogen atmosphere at a heating rate of 3 °C·min^-1^. **(B)** Differential scanning calorimetry (DSC) curve of MenX PS obtained under nitrogen atmosphere at a heating rate of 2 °C·min^-1^. **(C)** TGA/DTG curves of MenA PS obtained under nitrogen atmosphere at a heating rate of 3 °C·min^-1^. **(D)** Comparative TGA curve analysis of MenA and MenX PS.

DSC was used to monitor the thermal transition properties of MenX PS across temperature gradients. A broad exothermic event was observed from -50 to 170 °C, which corresponded to the disruption of molecular ordered arrangements and temperature-induced physical state variations (44). A sharp endothermic peak emerging at 170–300 °C indicated the occurrence of intense and continuous thermal degradation. Notably, this endothermic DSC signal was highly consistent with the second weight loss stage in the TGA/DTG profile, confirming that the thermal decomposition within this temperature range was predominantly driven by endothermic covalent bond cleavage, especially the breakage of glycosidic linkages (**Figure 5B**).

Previous literature has reported that MenA PS is more susceptible to hydrolytic degradation than MenX PS. The different stereochemistry of the N-acetyl group at position C_2_ of mannosamine (MenA PS) and glucosamine (MenX PS) might play a fundamental role in this susceptibility to polysaccharide chain degradation (31). Purified polysaccharides represent essential starting materials for glycoconjugate vaccine manufacturing and are routinely stored in solid form. To quantitatively compare solid state thermal profiles of the two polysaccharides and elucidate the correction between storage morphology and thermodynamic behavior, TGA/DTG was conducted on MenA PS with a standardized heating program. MenA PS displayed three sequential weight loss stages analogous to those identified for MenX PS (**Figure 5C**). Side-by-side comparative analysis failed to detect a distinct plateau following the initial weight loss stage of MenA PS, indicating mild thermal backbone degradation occurred simultaneously with the volatilization of residual water and solvents. MenX PS possessed a thermal stability threshold of 170 °C, whereas MenA PS initiated thermal decomposition at a temperature as low as 125 °C. In addition, MenA PS exhibited a substantially accelerated weight loss rate within the second stage, indicative of more aggressive thermolytic cleavage of glycosidic linkages. (**Figures 5A, C, D**).

### 3.6 Antigenicity and immunogenicity analysis of Neisseria meningitidis serogroup X polysaccharide

Antigen specificity represents a core quality attribute of polysaccharide antigens, ensuring precise and targeted immune activation. Immunodiffusion assays were adopted to analyze the antigen specificity of three consecutive batches of MenX PS produced under identical manufacturing processes, aiming to evaluate the specific immunoreactivity of the MenX PS against the target antiserum and exclude potential cross-reactions with other meningococcal serogroups. As illustrated in **Figure 6A**, the agarose gel matrix was arranged in a hexagonal pattern with central and peripheral wells, each loaded according to the predefined experimental scheme. Double immunodiffusion results demonstrated that all three MenX PS batches formed distinct and specific immunoprecipition arcs exclusively with homologous anti-MenX antiserum. No non-specific diffusion or cross-reactive precipitates were observed with heterologous antisera or saline negative control groups (**Figure 6A**). These findings confirmed that purified MenX PS prepared under the established process presents intact, and serogroup-specific antigenic epitopes that can be accurately recognized and bound by matching antiserum without detectable cross-reactivity. Subsequently, single radial immunodiffusion was employed for semi-quantitative analysis of specific antigen epitope content across the three batches. In this assay, antigens diffuse homogenously within antiserum-containing agarose gels to form circular precipition rings, whose diameters correlate logarithmically with antigen concentration (46). Under strictly controlled loading and diffusion conditions, no statistically significant differences in precipition ring diameters were observed among the three batches (**Figure 6B**), demonstrating excellent batch-to-batch consistency in specific antibody-binding epitope content.

**Figure 6:**
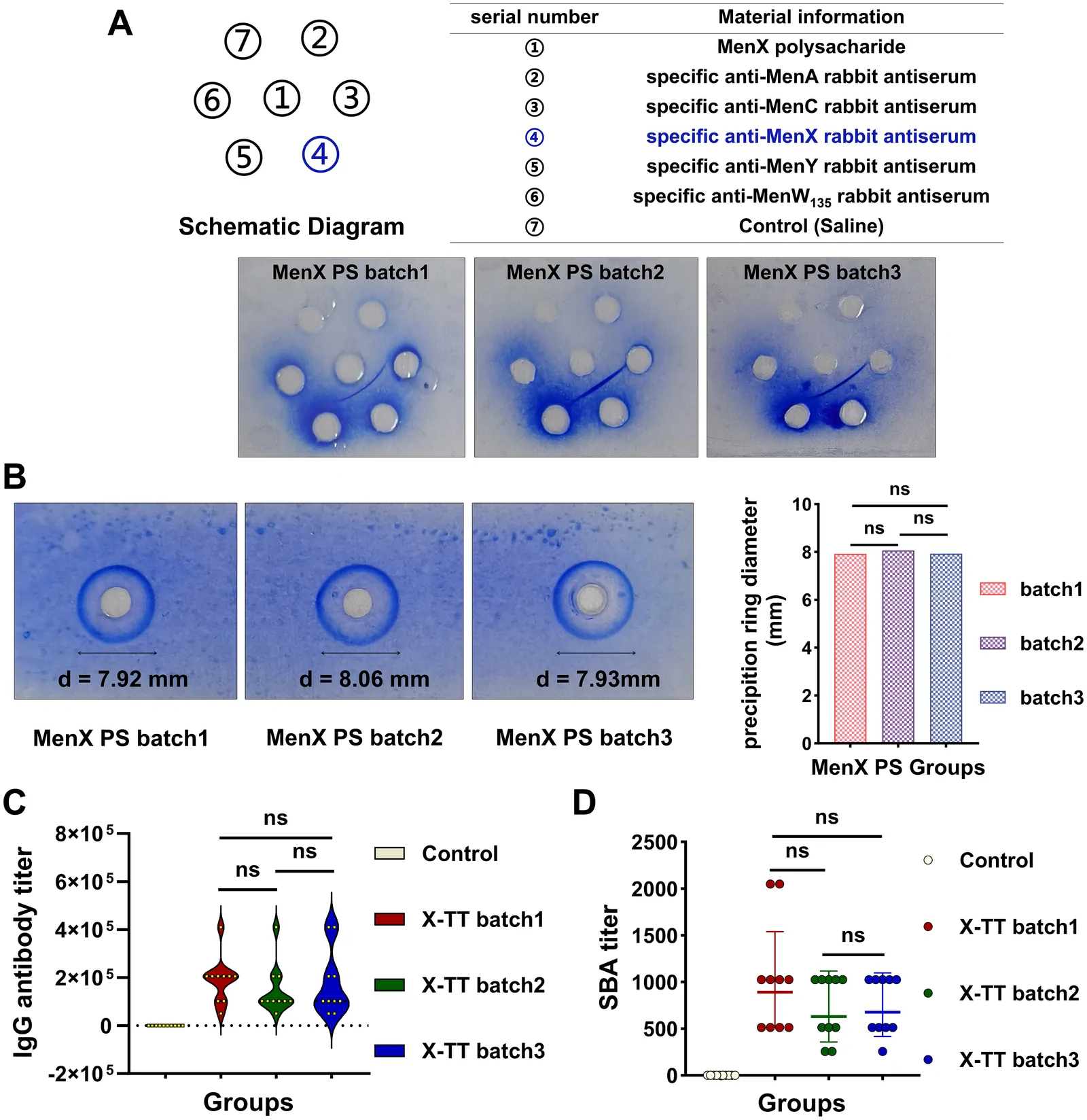
Antigenicity and immunogenicity analysis of MenX PS and X-TT conjugates. **(A)** Double immunodiffusion assay. Upper left panel: Schematic diagram of the double immunodiffusion assay, upper right panel: well-loading scheme for the agarose gel matrix, lower panel: immunodiffusion results of three batches of MenX PS. MenC: *Neisseria meningitidis* Serogroup C, MenY: *Neisseria meningitidis* Serogroup Y, MenW_135_: *Neisseria meningitidis* Serogroup W_135_. **(B)** Single radial immunodiffusion results of three MenX PS batches. Left panel: representative precipition rings formed with specific anti-MenX rabbit antiserum, right panel: statistical analysis on the diameters of precipition rings. ns indicate no significant difference. **(C)** MenX PS-specific serum IgG binding antibody titers in ICR mice following two-dose subcutaneous immunization with X-TT conjugates (n=10). **(D)** Serum bactericidal antibody (SBA) titers against the MenX CMCC 29040 strain induced by two-dose X-TT immunization (n=10). All data are presented as the geometric mean titer (GMT) ± standard deviation (SD), ns indicate no significant difference.

To further systematically evaluate the *in vivo* immunogenicity of MenX PS and verify its feasibility as an effective antigen component for MMCVs, three batches of MenX PS-tetanus toxoid conjugates (X-TT) were synthesized via chemical conjugation (47, 48). After completing antigenic profile validation (**Supplementary Figure 5**), ICR mice were selected as the primary animal model for immunogenicity assessment. Specifically, a two-dose immunization schedule was adopted for animal experiments to analyze the level of humoral immune responses and functional antibody activities elicited by X-TT vaccination. Serological assays demonstrated that all three batches of X-TT were capable of inducing robust serogroup-specific immune responses in immunized mice, with a 100% seroconversion rate for MenX PS-specific IgG binding antibodies. Geometric mean titers (GMTs) of endpoint binding antibodies were consistently higher than 120,000 across all batches (batch1 GMT = 165,959; batch2 GMT = 125,893; batch3 GMT = 134,896) (**Figure 6C**). The findings indicated that X-TT could elicit high levels MenX PS-specific IgG antibody in all immunized individuals, and also illustrated the prominent advantage of the TT protein in potentiating humoral immune responses against polysaccharide antigens (49–51). Serum bactericidal antibody (SBA) activity serves as the gold-standard functional indicator for evaluating vaccine-induced protective immunity, reflecting the *in vitro* bacterial clearance capacity of immune sera against live meningococci. Accordingly, a serum bactericidal assay targeting the MenX CMCC 29040 strain was established in accordance with international methodological guidelines to quantify functional bactericidal antibodies in mouse sera. SBA measurements revealed that X-TT elicited potent SBA responses, with 100% of immunized animals exhibiting SBA titers above the accepted protective threshold (≥1:8) (batch1 GMT = 891; batch2 GMT = 630; batch3 GMT = 675) (52) (**Figure 6D**). This result further confirmed that the induced antibodies possessed both specific antigen-binding capacity and potent biological function, which constituted the fundamental immunological basis for *in vivo* protective immunity against serogroup X meningococcal infection.

## 4 Discussion

This study systematically elucidated the key biological characteristics and underlying process control mechanisms of the MenX CMCC 29040 strain during industrial-scale fermentation for vaccine manufacturing. Genomic analysis confirmed the genetic stability of the CPS biosynthesis gene cluster across sequential fermentation generations, which guaranteed consistent antigenic epitope profiles and provided a molecular basis for vaccine quality control. A prominent innovation of this work lies in the pioneering application of TEM to dynamically monitor real-time changes in bacterial capsular thickness throughout the entire fermentation process (53, 54). That technical breakthrough converted the conventional “black-box” fermentation process into a visualizable, quantifiable system, and directly demonstrated the synchronous correlation between capsular development and bacterial growth dynamics. Elucidation of the capsular fermentation kinetic mechanism will enable precise optimization of fermentation feeding strategies to enhance final yield of target polysaccharides. Combined with SEM morphological observations, we further validated that the currently adopted parameters, including fermentation conditions, formaldehyde inactivation, and centrifugation, efficiently promote CPS shedding while preserving intact bacterial cellular integrity. The present findings possess both practical and theoretical significance. Practically, formaldehyde inactivation treatment increases polysaccharide yield (55) by inducing partial capsular dissociation rather than non-specific cellular lysis. Theoretically, this work uncovers the dynamic assembly properties of CPS within viable meningococcal cells. Formaldehyde induced capsular dissociation, as evidenced by reduced capsular thickness and increased abundance of free globular polysaccharide particles in aqueous media, suggests that the chemical inactivating agent disrupted capsule anchoring sites or interchain non-covalent interactions. This discovery offers novel mechanistic insights into the molecular interactions between chemical inactivating agents and bacterial capsular structure. Based on the above findings, future research will focus on elucidating the precise molecular mechanism of formaldehyde mediated capsular dissociation, investigating polysaccharide release kinetics, and defining the optimal harvest time point. Such efforts will deepen fundamental knowledge of meningococcal capsular biology and elevate the technical efficiency of industrial vaccine production pipelines.

More importantly, this study comprehensively verified the structural characteristics of MenX PS through multi-dimensional analytical approaches, further complementing the validated genetic stability of the production strain. Combined NMR and FTIR analyses confirmed that the chemical structure of MenX PS was consistent with previously reported canonical structures and lacked *O*-acetylation modification. This structural feature aligns with the genetic profile of the CMCC 29040 strain, whose *cps* locus is devoid of the *O*-acetyltransferase encoding gene (28, 30). Congo red staining assay (37) demonstrated that the absence of triple-helix structure in aqueous solution of MenX PS, confirming high molecular flexibility that supports the overall random-coil conformation visualized by TEM. Previous studies have reported that meningococcal CPS covalently anchored to the outer membrane and radiates outward from multiple bacterial surface anchoring sites. These anchored high-molecular-weight polysaccharide chains intertwine to form a mesh like capsular network that can be observed via TEM (56). In contrast, individual chains of purified MenX PS predominantly adopted a compact random-coil morphology in aqueous solution with potential helical structural segments (57). This unique molecular conformation is synergistically driven by multiple physicochemical factors. Glycosidic bonds provide sufficient dihedral angle rotational freedom for chain flexibility. Water-polysaccharide hydrogen bonding promotes solvation and attenuates interchain aggregation, while intramolecular hydrogen bonds dynamically balance electrostatic repulsion from negatively charged phosphodiester groups. Thermodynamically, conformational entropy favors coiled conformations over fully extended chain structures. Of course, future investigations should be done to elucidate the exact molecular mechanism underlying the compact globular random-coil conformation of free MenX PS. Furthermore, electron microscopy provided the first direct nanoscale visualization of the morphological traits of MenX PS. The high consistency between solid state SEM and solution state TEM observations excludes sampling-induced artifacts, verifying the robustness and reliability of the entire characterization workflow. Notably, quality-qualified MenX PS exhibited a predominant particle size distribution ranging from 100–200 nm. This unique dimensional size range endows favorable thermodynamic stability and falls within the preferred size window for antigen uptake by antigen-presenting cells (APCs), which benefits the induction of potent immunogenicity (58). Meanwhile, the prominent surface curvature, distinct roughness, and porous topology of MenX PS particles may modulate their biological performance through multiple physicochemical mechanisms. The integrated analytical strategy, combining traditional spectroscopic identification with advanced nanoscale morphological characterization, is expected to facilitate the development of MMCVs from empirical exploration to rational design. This methodological paradigm provides a referable framework for the structural and functional evaluation of other bacterial polysaccharide vaccine antigens.

Another significant contribution of our work lies in the utilization of TGA/DTG analytical techniques, which upgrades the evaluation of thermal stability for MenX and MenA PS from qualitative observation to quantitative and mechanism-driven interpretation. The solid state thermal degradation thresholds of both polysaccharides were precisely quantified, supplying comprehensive data for direct comparison of their thermal performance. The experimentally identified critical thermal threshold of 170 °C for MenX PS serves as a fundamental parametric foundation for deploying the Quality by Design (QbD) principle throughout vaccine development and offers reliable reference for setting the temperature parameters of total solids assays. Distinct multistage weight loss profiles captured by coupled TGA/DTG analyses corroborate that MenX PS undergoes an ordered, stepwise thermal degradation pathway. Follow-up experiments integrating TGA with mass spectrometry (MS) or FTIR will permit *in situ* identification of volatile degradation intermediates released at discrete thermal decomposition stages. Such complementary characterizations will establish direct correlations between weight loss events and specific chemical reactions, yielding high-resolution mechanistic insights into the thermal degradation of MenX PS at the molecular level.

Previous studies have indicated that polysaccharides with low molecular weight exhibit poor immunogenicity in humans (59, 60). Based on our experimental data and the purification strategy described by Yang and Jennings (34), we rationally designed and optimized the purification pipeline to produce MenX PS with an Mw of 317,500 g.mol^-1^, corresponding to a molecular size range favorable for eliciting potent immunogenic responses. Antigenicity analysis confirmed that purified MenX PS retained native conformational epitopes capable of inducing serogroup specific antibodies without detectable cross-reactivity against other prevalent meningococcal serogroups, establishing a high-quality antigenic foundation for glycoconjugate vaccine development. Conjugation, covalently coupling the CPS to carrier, changes the human immune response from T-cell independent to T-cell dependent engaging helper T cells in maximizing a predominant high affinity IgG immune response (48, 61). Conjugation ultimately results in an improved primary antibody response, especially in young children, creates affinity maturation, immunologic B cell memory and an amnestic IgG response at re-exposure (62, 63). Bacterial CPS represent promising vaccine antigens that can be conjugated to multiple carriers, such as outer membrane vesicles (OMVs) (64) and proteins (47), to develop prophylactic vaccine candidates. Although biosynthetic strategies for conjugate vaccines enable the production of vaccine candidates against diverse pathogens, such as Vibrio cholerae, Salmonella paratyphi A, extraintestinal Escherichia coli, and Brucella spp., these approaches are mainly applicable to O-polysaccharide (OPS). To date, biosynthetically engineered CPS conjugates have been successfully developed for a limited range of pathogens, and the efficient conjugation of CPS with proteins remains an unresolved technical challenge (61, 63, 65–67). Therefore, this study adopts more mature chemical conjugation approach to accelerate the clinical translation of glycoconjugate vaccines. X-TT conjugate synthesized via reductive amination chemical conjugation process elicited potent humoral immune responses in mice without observable adverse effects. Following a two-dose immunization regimen, the X-TT conjugates exhibited high titers of MenX PS-specific total IgG and functional bactericidal antibody induced in mouse models, meeting the core immunological evaluation criteria for vaccine candidates (62, 68). Given the challenges in the clinical translation of complex biological formulations for MMCVs (23), subsequent investigations will focus on evaluating formulation compatibility and immunological synergism between X-TT and glycoconjugates of other meningococcal serogroups. Critically, appropriate quality control analytical tools will be created for these complex biological formulations to generate systematic preclinical data that guide the rational formulation design of multivalent vaccines. Notably, although our candidate X-TT conjugates exhibited non-inferior immunogenicity relative to the MenX-TT component of the licensed NmCV-5 vaccine (Serum Institute of India) (68), the animal experimental design of this study has inherent limitations in parallel control setup. The only commercially licensed NmCV-5 vaccine and its matched single MenX-TT conjugate reference component are inaccessible via standard compliant research channels. Therefore, the mouse immunization model established herein was primarily designed to evaluate the batch-to-batch immunogenic consistency of three independently manufactured X-TT conjugate batches. Future preclinical studies will incorporate the aforementioned missing control groups to enable comprehensive and rigorous assessment of the immunological potency of candidate MMCVs containing the MenX PS antigen component.

Collectively, our work validated the robustness and controllability of the complete manufacturing pipeline, including bacterial cultivation, polysaccharide extraction, and purification. Comprehensive physicochemical characterization of MenX PS and *in vivo* immunogenicity evaluation of X-TT conjugates in mouse models further support that MenX PS as a promising antigen for MMCVs. These findings lay a critical foundation for the large-scale manufacturing, quality control and preclinical development MenX PS-based conjugate vaccines.

## Data Availability

All relevant data is contained within the article. The original contributions presented in the study are included in the article/**Supplementary Material**, further inquiries can be directed to the corresponding authors.
