# Supplementary material for: Neisseria meningitidis serogroup X polysaccharide as a qualified antigen for multivalent meningococcal conjugate vaccines
Source: Front Immunol. 2026 Oct 8;17:1943863. doi: 10.3389/fimmu.2026.1943863 (PMC13686849; doi:10.3389/fimmu.2026.1943863)
Supplement: Supplementary file 1 [file DataSheet1.pdf]

Supplementary Materials

1 Supplementary Figures and Tables

1.1 Supplementary Figures

Supplementary Figure 1. Phenotypic characterization and taxonomic identification of the *Neisseria meningitidis* serogroup X (MenX) CMCC 29040 strain during fermentation. The analysis includes master seed (M), primary working seed (W0), and final fermentation seed (W4). The performed assays encompass cultural characteristics at 25 ℃ and at 35–37 ℃ with 5–10% CO_2, Gram staining, biochemical reaction (carbohydrate utilization), and serological confirmation by tube agglutination.

Supplementary Figure 2. Genetic stability analysis of capsular polysaccharide (CPS) translocation genes in MenX CMCC 29040 strain during fermentation. Sequence alignment and homology assessment of CPS translocation genes in Region B. MenX_M: master seed, MenX_W0: primary working seed, MenX_W3: 30 L-scale fermentation, MenX_W4: 100 L-scale fermentation.

Supplementary Figure 3. Genetic stability analysis of CPS transport genes in MenX CMCC 29040 strain during fermentation. Sequence alignment and homology assessment of CPS transport genes in Region C. MenX_M: master seed, MenX_W0: primary working seed, MenX_W3: 30 L-scale fermentation, MenX_W4: 100 L-scale fermentation.

Supplementary Figure 4. ^{31}P NMR and two-dimensional NMR spectroscopy of MenX polysaccharide (MenX PS). The figure presents the ^{31}P NMR spectrum and the following two-dimensional spectra used for structural elucidation: ^{1}H-^{1}H COSY (homonuclear correlation spectroscopy), ^{1}H-^{13}C HSQC (heteronuclear single-quantum coherence), and ^{1}H-^{13}C HMBC (heteronuclear multiple-bond correlation) .

Supplementary Figure 5. Single radial immunodiffusion results of three batches of MenX PS-tetanus toxoid (X-TT) conjugate. (A) Representative precipition rings formed with specific anti-MenX rabbit antiserum. (B) Statistical analysis on the diameters of precipition rings. ns indicate no significant difference.

1.2 Supplementary Tables

Supplementary Table 1. The analytical information for three batches of MenX PS

Sample information	Phosphorus content (mg/g)	Protein content (mg/g)	Nucleic acid content (mg/g)	Endotoxin (EU/μg)	molecular size distribution	
					K_D	eluted under the main peak
MenX PS batch1	83.75	4.21	1.99	3.00	0.28	91.10%
MenX PS batch2	90.49	4.11	2.67	3.03	0.24	90.20%
MenX PS batch3	82.49	4.89	3.04	0.88	0.27	93.5%

All polysaccharide samples used in this study were obtained from three qualified batches of MenX PS listed in the table, which comply with WHO Recommendations[1-3] and the European Pharmacopoeia (2025) for purified bulk polysaccharide for use in vaccines.

Supplementary Table 2. The critical analytical information for three batches of X-TT conjugate

Sample information	MenX PS/TT (wt/wt)	Endotoxin (EU/μg)	molecular size distribution	
			K_D	eluted under the main peak
X-TT batch 1	0.51	0.40	0	89.70%
X-TT batch 2	0.59	0.20	0	90.00%
X-TT batch 3	0.56	0.10	0	91.30%

Supplementary Table 3. Gene sequences involved in CPS biosynthesis within the *cps* locus of MenX CMCC 29040 strain

Gene Region	Gene Name	Gene Sequence
	csxA	atgattatgagcaaaattagcaaattggtaacccacccaaaccttttctttcgagattatttc ttaaaaaaagcaccgttaaattatggcgaaaatattaaacctttaccaatcgaaacctctt ctcatagcaaaaaaaatacagcccataaaacacccgtatcatccgaccaaccaattgaa gatccatacccagtaacatttccaattgatgtagtttatacttgggtagattcagatgatga aaaattcaatgaagaacgcctaaagtttcaaaattcaagcacatctgagactctacaagg caaagcagaaagcaccgatattgcaagattccaatcacgcgacgaattaaaatattcga

Region A		ttcgaagcctgatgaagtatgccccatgggtaaatcatatttacattgtaacaaatggtca<br>aataccaaaatggttagataccaacaatacaaaggtaacgattatccctcactcaactatt<br>atcgacagtcaatttctccctacttttaattctcacgtcattgaatcctctctatataaaatcc<br>caggattatcagagcattacatttatttcaatgatgatgtcatgctagctagagatttaagc<br>ccatcttatttctttacaagcagcggattagcaaaactgtttattaccaactctcgtctacca<br>aatggctataagaatgtgaaagacacaccaacccaatgggcctcaaaaaattcccgtg<br>agcttttacatgcagaaacaggattttgggctgaagccatgtttgcacatacttttcatcca<br>caacgtaaaagtgtacatgaatctattgaacacctatggcatgaacaattaaatgtttgtc<br>gtcaaaaccgtttccgtgatatttcagatattaacatggcgacattcctgcaccaccatttt<br>gccattttgacaggccaagctcttgctacacgcactaaatgtatttactttaacattcgctct<br>cctcaagcagctcagcattacaaaacattattagctcgaaaaggaagcgaatacagcc<br>cacattctatctgcttaaatgatcatacatcgagcaataaaaatattttatctaattacgaag<br>ccaaattacaaagctttttagaaacatactatccagatgtatcagaagcagaaattctcctt<br>cctactaaatctgaagtagctgaattagttaaacataaagattatttaactgtatatactaaa<br>ttattacctattatcaataagcagctggtcaataaatataataaaccttattcatatcttttcta<br>ttatttaggtttatctgcccggtttttatttgaagaaacgcaacaagaacactaccgggaa<br>actgctgaagaaaatttacaaatcttttgtggcctaaacccaaaacatacactagccctca<br>aatacttagcggatgtcaccctcacatcacagcctagtggacaataa
	csxB	atgtcaccctcacatcacagcctagtggacaataataatctaagttacggagtaatcgtat<br>ttatgaatactatacaaaactatccagcatatcaaaatgatacagatattgcaaagcaaaa<br>aacttcgtacactgaattaggggttaattttaatccggattattctaaagtacgtcttgtatta<br>caaacaaatagtcgcttaaacattattaaattagcccgaagagatgcccaagcttacgat<br>caatatattgaaattataaaaaatgattttattttaattagtcatcatcgtgatggctattcagt<br>atttatgaaacgtaaggagtaccatccggagcgactgaaggcttacagcgatttgcgcc<br>gccataaagaaatgttatacacactgacaccaccttcaccaaaacgcaaatctgcctct<br>gtccccaaccgtttattggtattgtttgcccacatgaatggcggtggcggctatgatagc<br>agcaatgtcgttgagcgattatttgtccaatatttcgcagatgttcaacgcagcttggtca<br>aaaacgtctatgtactgcgcattgctgatttgaatatctcccatggctcttactttaccaata<br>ctgcaaactatccggattacgaagaccagattcagagcctgattaaagaaaccttatac<br>aaatgcgacatccagcctgaaaacgtcgttttatatggaggcagtaaaggtggcgcgg<br>gggcactattacatggtgccattggcaactacaaagtggtagccggcgacccaattatt<br>gactcatcggtttataatcaaaaagatgtccatttcgtcaaacattttaaagaagccaacat<br>gactaacatgattgttaatcatctacgcggcaatactcataaaatgtatatttgcgcatcta<br>gtgcacagaaattcaattttgaaacaagttctaatctagctcgcgcctcgagcggattaat<br>caacattgttgacttatcgaatgacatacagattaagacgcaccccaaattactgctcact<br>ccgtccctgaacagataa
	csxC	atgaaaaaatttttttctatcctctcgatatttatcttatctttctttaactctgaatacacatacg<br>ctcaatctttgtgtattcagcaaagctcacaaaaccatatccatatcgcaaaaatcaactta<br>aattgtaaaggtatcaatttaatcgccacccaagaagccgacaaaggcatgacagtctc<br>tcaatttgcccgtaaataccgtactgatatcgcgattaatggtagtttttttccgcaccggat<br>atttcccttttggcctcgccattaccgatcataaaacttgggacaaaacccgcgatgtcca<br>aaaacgggttttttttagcctgtaatcggcaaaatcgctgtatgatcgaagataaaaatatg<br>gtttctaaagtagatgattcttggaaactagccgtatccggctggcaatcttttaatccgg<br>caaccaaaaaatttgaatgttccgatgatgatccggttggttgtacgcatattaaatttata

		acgaaacaacctcgcacgatggttggattagacgaaaagcgtaattatttatactttgtcg<br>tgatagatggcaggctgcctaaatttaaaggtgctactttaaatgaattaggccaattagc<br>cgcaagcctcaaattaactaaggccattaatttagatggcggcggcagctctactatggt<br>caagggatataaccgtatatcgaccttacccgccacacaaaaaaaagaacgtgtggta<br>gcgaatcatttgggcgtaaaatatatctctcgataa
Region B	*ctrE*	atgttcctcttttcagacggcctacaatctattaataacaataatcgacgaaaacgcattgt<br>gaaaaacgcctacatcccctctcgcggcatccgcaaaatcccccatctctccaccctatt<br>gcccgagtttcatatctacaaagacggaaaaggagcagaggctgttgtcggctggggt<br>ttgcgcccgacgacacacaaagcgcgtgcttttgccaccgaacaccagcttccctttatt<br>gctttggaagacggctttttacgctcgctcggactgggcgtctccggttatccccctac<br>tccatcgtctatgacgacatcggcatctactacgacactacccgcccttctcgtttggaac<br>aactgattcttgccgccgataccatgccgtctgaaaccttggctcaggcgcaacagacg<br>atggatttcatcctgcaacaccacctgtccaaatacaaccacgcgcccgaactttcaga<br>cgatcatcctttacgctccccatccaagcctgaaactgtcctcattatcgaccaaaccttc<br>ggcgatatggccatccaatatggcggcgcagacgcctctacgtttgaactgatgtttca<br>gacggccttaaatgaaaacccgcaagccgatctttgggtcaaaacccaccccgatgttt<br>tgtgcggcaaaaaacaaggctatctgacccagctggcgcagcaacaccgcgtccatc<br>ttttggcagaagacatcaatccgatttctttgttgcaaaacgtcgataaagtttattgcgtta<br>cctcgcaaatgggttttgaggcgcttttgtgcggcaagccgctgaccaccttcggactg<br>ccgtggtatgccggatggggcgtgagcgacgaccgccacccaaaaatcgacagcctt<br>gttcaaacccaacgccgcatcccccgcaacttgctgcaactcttcgccgcagcctatct<br>gcaatacagccactacctcaatcccaataccggcgaagcaggcagcctctttgatgtca<br>tcgactatctggcgacgatcaaacgcaaaaacgacaaattgcgtggcgagttgtattgc<br>gtcggcatgtctttgtggaaacgcgcggttgccaaaccgttctttaacgtaccctcatgc<br>cgtctgaaatttatctcctccacacaaaaactggctagggtcaaactgtccgacgatgca<br>cgcatcctggcctggggcaacggtaaagaggccatcgtccgctttgctgaacaacaca<br>acatccccctgctgcgcatggaagacggctttatccgctcggtcggactcggctccaa<br>cttagtaccgccgctgtcgcttgttaccgacgatatgggcatttatttcaatgccgaagcc<br>ccatcccgccttgaacacatcctgcaaaaccaaaacttcgacgatcaagactttcagac<br>ggccttgaagttgcaaaaaatgctgaccgaaaaccatatcagcaaatacaacgtcggc<br>aactcagactttaccgccccatcaaccgacaaaaccgtgatcctcgttcccggtcaggt<br>tgaggatgatgcgtctatccgctatggttcgccccaaatctatcacaatctggatttgctc<br>cgtaccgtacgcgaacgaaaccccaatgcctatatcatctacaaaccgcatcccgatgt<br>agtcagcggtaaccgcatcggccatatttcccctgaagatgctgcacgatatgccgacc<br>aaaccgccgaacaagccgacatcctgacctgtctccaatacgcagacgaaatacatac<br>catgacttcgctgaccggttttgaagccttgttgcgcggcaaaaaagtcagctgctacg<br>gcctgcctttttacgcaggctgggggcttacccaagatctgctccccatcccgcgccgt<br>agccgcagacttgagctttggcagctgattgccggcacgctcatccactatcccgacta<br>catccaccccgaaacccatcaggccataaatgcagaaaccgcagcccaaatcctgat<br>acgacaaaaaaatatgcaaaaaaacaacaacggattacatcgcgggtactttgccaaa<br>aaattaggtaaaatcaaacaactatatcgatctttcaaataa
	ctrF	atgaaacagaccgtcctcaaaaataacctgcaaaacctgcttgaaagcgcagaaaata<br>tcctgctgcttcaaggccctgtcggcgatttttttctgcgccttgccgactggctgactgc<br>aaacggcaaaaccgtacataaattcaactttaatgcaggcgacgactatttttatccgcc

		cactcaagcgcataccgttgtttttaacgacaactacgatgcctttcctgagtttttgcaag<br>aatacatcactcaacatcacatccaggccgttgtctgctttggcgacacacgcccttatc<br>acgtcattgcaaaacgcattgcaaacgaaaaccaagccagtttctgggcgtttgaagaa<br>ggctatttccgcccctactacatcaccttagaaaaagacggcgtcaacgcattttccccg<br>ttgccgcgccgtgccgactttttcttgaacaattccctaagcttgcccagcaagaatata<br>aagcgccaacgccggtacacggcggttttacgcccatggcaaaaaacgctatccgtta<br>ctatatcgagttgttccgcaatccacgcaaataccccgactacatccaccaccgcgcac<br>ccaatgccggccattacctcaaaccgtggtcgctctccatcctcaagcgtttgaactact<br>atattgaagacatccaaatcgccaaacgtgtggaagcaggcaaatacggcaagtttttt<br>attgttcccttacaggtattcaacgacagccaagtccgtatccattgcgactttcccagcg<br>tccgcagcttcctgctccatgttttgagttcatttgccgagcacgcgcctgccgataccaa<br>catcatcatcaagcatcatcctatggaccgcggctttatcgactactggcgcgacattaa<br>acgctttatcaaagaacaccccgaactcaaaggccgtgtgatttatgtccatgatgtccc<br>cctgcccgttttcctgcgccacggtctcggcatggttaccatcaacagcaccagcggcc<br>tgtccggactgattcacaatatgccagttaaggttctcggccgtgcctattatgatattccc<br>ggtattactgaccaaaataccttggcagaattttggaatcatccgacaccgcctgacaaa<br>gagctgttccatgcctaccgaatgtaccacctcaacgtgacccaaattaacggcaactt<br>ctacagtcaggtctttttttcctaacaaaaaaacctccgactcttccactccagcaacctga
	ctrA	atggttatgcaaattaaatgcaaattcaattggttggtacttatgaccgctacaatggttgt<br>gggttgtagttctttgccaacaacagggccgagtgctaagaaagtagaatctttaagtca<br>ggtatcttctgaaactcaaatccctgaagtggaattgattgatgtcaaccatgctgtggcg<br>cagtctttgtataaggcccaagtaaatcagtccttcactcagtttggcgatggttatgcttc<br>ggctggtacgctaaatattggtgatgtattggatattatgatttgggaagcgccgccggc<br>agtattgtttggtggtggcctttcttcgatgggctcgggtagtgcgcatcaaactaagttg<br>ccagagcagttggtcacggcacgtggtacggtttctgtgccgtttgttggcgatatttcg<br>gtggtcggtaaaacgcctggtcaggttcaggaaattattaaaggccgcctgaaaaaaat<br>ggccaatcagccacaagtgatggtgcgtttggtgcagaataatgcggcgaatgtgtcg<br>gtgattcgtgctgggaatagtgtgcgtatgccgctgacggcagccggtgagcgtgtgtt<br>ggatgcggtggctgcggtaggtggttcaacggcaaatgtgcaggatacgaatgtgca<br>gctgacacgtggcaatgtagtacgaactgttgccttggaagatttagttgcaaatccgcg<br>acaaaatattttgctgcgtcgcggtgatgtggttaccatgattaccaatccctatacctta<br>cgtctatgggtgcggtggggagaacacaagaaatcggtttttcagccagaggcttatc<br>gctttctgaagccattggccgtatgggcggtttgcaagatcgccgttctgatgcgcgtg<br>gtgtgtttgtgttccgctatacgccattggtggaattgccggcagaacgtcaggataaat<br>ggattgctcaaggttatggcagtgaggcagagattccaacggtatatcgtgtgaatatg<br>gctgatgcgcattcgctattttctatgcagcgctttcctgtgaagaataaagatgtattgtat<br>gtgtcgaatgcgccgttggctgaagtgcagaaatttttgtcgtttgtgttctcgccggttac<br>cagtggcgcgaacagtattaataatttaactaattaa
		atgtctgagcaacttcctgtggcagttgccactgaaaccaaagccgagcgtaaaaagc<br>cgaaaaagaaaagttggattaaaaagctaagccctttattttgggtaacggtgattatccc<br>tacggtaatttcgttggtgtatttcggcttcttcgcttccgatcgttttacgtcgcaatcgag<br>ctttgtggtgcgctcgcctaaaagccaatcttctctcaatggcctgggtgccattttgcag<br>ggcacaggttttgcccgtgcgcaagatgatatttacacggttggggagtatatgcgttcg<br>cgctcgtctttggatgaactgcgtaaaatcttgccggtgcgtgagttttatgaaaccaaa

Region C	*ctrB*	ggtgatgcgttcagccgctttaatgggtttgggttccgtggcgaggaagaggctttttat<br>caatactataaaaatcaggtgatgatcaattttgatacggtttcgggtatttccacgttgaa<br>tgtaacttcctttgatgcgctggaatctaagaaaatcaatgaggctttgttaaaacaaggt<br>gaagcattgattaaccagttgaacgatcgtgcacgtgctgatacggtgcgctatgcgga<br>agaagtagtgaaaacggcggcagagcgggtaaaggaagcctctcagaatctgacgg<br>attaccggattgccaatggcgtttttgatttgaaagcgcaatcggaagtgcaaatggggt<br>tggtttccaagctgcaagatgaattgattgtgattcaaacccagctggatcaggtgaaag<br>cagtcactccggagaatccgcagattccgggtttgcaggcgcgtgagcagagcttgc<br>gtaaagaaattgaccaacagttacgtgccatttcgggcggtgggcattcttcgttgtcta<br>atcaggctgccgaatatcagcgtgtgtatttggaaaaccagttggcagagcagcagttg<br>gcagccgccatgacttctttggaaagtgccaaggttgaagcagaccgtcagcagcttta<br>tttggaagtgatctcgcaaccgagcctgccggatttggcacatgagcctaaacggttat<br>acaacattgttgccactctgattatcggcttgatggtttatggtattttgagcctgttgactg<br>ccagcattcgtgagcataaaaactga
	ctrC	atgaaagccttgcataaaacatcattttgggaatctttagccattcaaaggcgcgtaatcg<br>gtgcgctgttgatgcgggaaattatcacccgttacggtcgcaataatattggctttttatg<br>gctgtttgttgagccgttgctgatgacattcgttatcgtcttgatgtggaaatttttaagggc<br>agaccgatattcaactttgaatattgtcgcatttgcgattactggctatccgatgttgatgat<br>gtggcgtaatgcctcaaaacgggcagttgggtcgatttcttcaaatgccagcttgctttat<br>caccgcaatgtaagagttttggataccatcttggcgcgcatgattttggaaattgctggtg<br>caaccattgcgcagattgtgattatggcggtattgattgcgattggctggattgaaatgcc<br>ggcagatatgttttatatgctgatggcttggcttttgatggctttttttgcgattggtttgggtt<br>tggtgatttgttcgattgcctttaatttcgagccgtttggcaagatttggggcacattgactt<br>ttgtgatgatgccgttatccggtgcgttctttttgtgcataatttgccgcccaaggtacaa<br>gaatatgcattaatgattccgatggtgcatggcacagaaatgttccgtgccggatattttg<br>gcagcgatgtaattacctatgaaaatccttggtatatcgtattgtgcaatctggtgttgttgt<br>tgtttggcttggcgatggtcagtaaattcagtaaaggagtcgagccgcaatga
	ctrD	atgatttcagttgaacacgtttccaaacgctatctgacccgccaaggttggcggacagtc<br>ttgcacgatattagcttcaaaatggagaagggcgagaaaatcggtattctcggccgcaa<br>cggtgcaggtaaatcgacgctcatccgtttgatcagtggcgttgagccgccgaccacg<br>ggtgaaatcaagcggacaatgagtatttcttggcctttggcattctccggtgcgtttcaag<br>gcagtctgaccggtatggacaatttgcgtttcatctgccggatttacaatgtcgatatcga<br>ttatgtgaagaactttaccgaagagttttccgagttggggcaatatctttacgagccggtc<br>aagcgttattcttcaggtatgaaggcgcgtttggcatttgctttgtcgttggcggtagagtt<br>tgattgctatctgattgacgaagtgattgcagttggcgactcgcgctttgccgataaatgt<br>aagtacgagttgtttgaaaagcgcaaagaccgttccatcatcttggtgtcgcacagcca<br>cagcgccatgaagcaatattgcgataatgcgatggtgctggaaaaagggcatatgcac<br>cagtttgaagatatggacaaagcctacgaatattataattcgctaccttaa

Supplementary Table 4. NMR chemical shift assignments (^{1}H, ^{13}C) for MenX PS repeating unit.

Position	δ ^{1}H NMR (ppm)	δ ^{13}C NMR (ppm)	δ ^{13}C DEPT153 (ppm)
1	5.58	94.07	94.07, CH
2	4.05	53.58	53.58, CH
3	3.96	69.95	69.95, CH
4	4.11	73.88	73.88, CH
5	4.02	72.03	72.03, CH
6	3.88	60.04	60.04, CH_2
7	2.09	22.05	22.05, CH_3
8	/	174.55	/

References

1 Vipond, C. *et al.* Evaluation of candidate international standards for meningococcal serogroups A and X polysaccharide. *Biologicals* **47**, 33-45, doi:https://doi.org/10.1016/j.biologicals.2017.03.001 (2017).

2 WHO. Requirements for meningococcal polysaccharide vaccine. WHO Technical Report Series 658. Geneva. 1981. https://www.who.int/publications/m/item/meningococcal-polysaccharide-vaccine-annex-6-trs-no-658.

3 WHO. WHO Recommendations for the preparation, characterization and establishment of international and other biological reference standards (revised 2004). WHO Technical Report Seriesvol. 932. WHO Expert Committee on Biological Standardization; 2006. p. 1-137. https://www.who.int/publications/m/item/annex2-trs932.
